# Exploring neural networks to uncover information-richer features for protein interaction prediction

**DOI:** 10.1007/s00249-025-01742-2

**Published:** 2025-04-03

**Authors:** Greta Grassmann, Lorenzo Di Rienzo, Giancarlo Ruocco, Edoardo Milanetti, Mattia Miotto

**Affiliations:** 1 Department of Physics, Sapienza University, Piazzale Aldo Moro 5, 00185 Rome, Italy; 2 Center for Life Nano & Neuro Science, Istituto Italiano di Tecnologia, Viale Regina Elena 291, 00161 Rome, Italy

## Abstract

Moving in a crowded cellular environment, proteins have to recognize and bind to each other with high specificity. This specificity reflects in a combination of geometric and chemical complementarities at the core of interacting regions that ultimately influences binding stability. Exploiting such peculiar complementarity patterns, we recently developed CIRNet, a neural network architecture capable of identifying pairs of protein core interacting residues and assisting docking algorithms by rescaling the proposed poses. Here, we present a detailed analysis of the geometric and chemical descriptors utilized by CIRNet, investigating its decision-making process to gain deeper insights into the interactions governing protein-protein binding and their interdependence. Specifically, we quantitatively assess (i) the relative importance of chemical and physical features in network training and (ii) their interplay at protein interfaces. We show that shape and hydrophobic-hydrophilic complementarities contain the most predictive information about the classification outcome. Electrostatic complementarity alone does not achieve high classification accuracy but is required to boost learning. Ultimately, our findings suggest that identifying the most information-dense features may enhance our understanding of the mechanisms driving protein-protein interactions at core interfaces.

## Introduction

Exploring the mechanisms underlying protein-protein binding is a fundamental goal in molecular biology, with significant implications for therapeutic advancements and biotechnology. For example, binding site prediction is critical in structure-based drug design.

Experimental methods like X-ray crystallography and NMR spectroscopy allow the detection of protein complexes but are often costly and resource-intensive (Miotto et al. 2024). Computational approaches provide a valuable alternative that offers cost-effective ways to simulate protein binding and capture dynamical structural and energetic shifts at an atomic scale over time. These insights are especially crucial for predicting interactions in the dense cellular environment, where proteins and other biomolecules coexist at high concentrations (Minton 1981; Minton and Wilf 1981; John Ellis 2001; John Ellis and Minton 2003; Zimmerman and Trach 1991). In such environments, specific binding interactions must be finely regulated to enable target recognition while disfavoring non-specific associations (Sheinerman and Honig 2002). Deciphering these binding mechanisms highlights the importance of computational models, which allow the integration of various molecular characteristics and contribute to the accurate prediction of biologically relevant protein interactions (Grassmann et al. 2024).

The identification of these components relies on the learning of effective molecular representations. The advent of Deep Learning (DL) methods has significantly enhanced the prediction of molecular properties. These machine learning algorithms use multiple layers to progressively extract higher-level features from raw input data, which can be derived from sequence or structural information. The importance of the latter has been growing since the introduction of AlphaFold2 (Jumper et al. 2021) and RoseTTAFold (Baek et al. 2021), which are able to predict 3D protein structures (with a resolution close to experimental data) directly from amino acid sequences.

However, incorporating all the features known to determine binding increases computational costs, making the prediction of interacting sites a difficult challenge. Given the already high cost of the numerous potential contact patches between two protein structures, developing efficient methods that focus on key interaction features is essential.

We recently introduced a novel method, the Core Interacting Residues Network (CIRNet), to assess the complementarity of various physical and chemical properties within an unified computational framework, with the aim of identifying interacting residues at the core of protein binding sites (Grassmann et al. 2024).

CIRNet identifies core interacting residues with a Receiver Operating Characteristic Area Under the Curve (ROC AUC) of 0.72 in a blind-search setting. When tested on three widely-used docking servers -ClusPro (Comeau et al. 2004), PyDock (Cheng et al. 2007), and LZerD (Christoffer et al. 2021)- CIRNet was able to improve the docking outcomes by rescaling the proposed poses.

The protocol is based on a compact representation of regions on the protein’s molecular and electrostatic potential surfaces through the Zernike 2D polynomials expansion. See the Methods section for more details.

We selected 2D Zernike polynomials due to (i) their efficiency in measuring shape complementarity, which correlates well with van der Waals interactions (Gainza et al. 2020) and binding affinity (Desantis et al. 2022). In 2021, we proposed a protocol based on this approach, showing that applying this feature alone in an unsupervised way could identify true interaction regions in approximately 60% of cases (Milanetti et al. 2021; Miotto et al. 2024). (ii) Unlike other structure-based methods that compute intermolecular atom-atom interactions directly, our approach uses a compact surface-based description to enable fast comparisons across potential interacting regions. Although 3D Zernike descriptors (Kihara et al. 2011; Daberdaku and Ferrari 2019; Zhu et al. 2015; Venkatraman et al. 2009) can also be used for such comparisons, the 2D Zernike descriptors are computationally faster. (iii) Furthermore, the Zernike descriptors are invariant under rotation, enabling compatibility assessments (via the Euclidean distance between Zernike vectors) without the need to roto-translate the protein in space to test possible orientations (Milanetti et al. 2021; Miotto et al. 2021; Bò et al. 2020; Grassmann et al. 2021; Lorenzo et al. 2021; Grassmann et al. 2022). (iv) Beyond shape, the Zernike approach is adaptable for any feature represented as numerical values on the protein surface, such as electrostatic potential. By integrating electrostatic complementarity within this framework, we recently demonstrated that it can distinguish between transient and stable protein complexes with an ROC AUC of approximately 0.8 (Grassmann et al. 2023).

To further refine binding characterization, CIRNet evaluates another crucial factor in protein binding: the hydrophobic and hydrophilic properties at binding interfaces (Rego et al. 2021). This is achieved by defining a hydropathy complementarity based on the product of residue hydrophobicity indices, as determined by a hydrophobicity scale that we developed (Di Rienzo et al. 2021).

To shed more light on the interplay between these features in determining binding, we identify the most important information for the successful learning of CIRNet. Shape and hydropathy complementarity were found to be the most informative features, even if electrostatic complementarity also improved learning. We did not observe clear correlations between these features, but CIRNet still picked up an interplay between them. Indeed, the NN-predictions are shifted to different ranges for various chemical classes. Thus, we tested if residue-specific thresholds can improve the accuracy of CIRNet in identifying interacting residue pairs, without modifying the input data space or the NN architecture.

## Results and discussions

### Complementarities of core interacting residues depend on their chemical nature

**Fig. 1:**
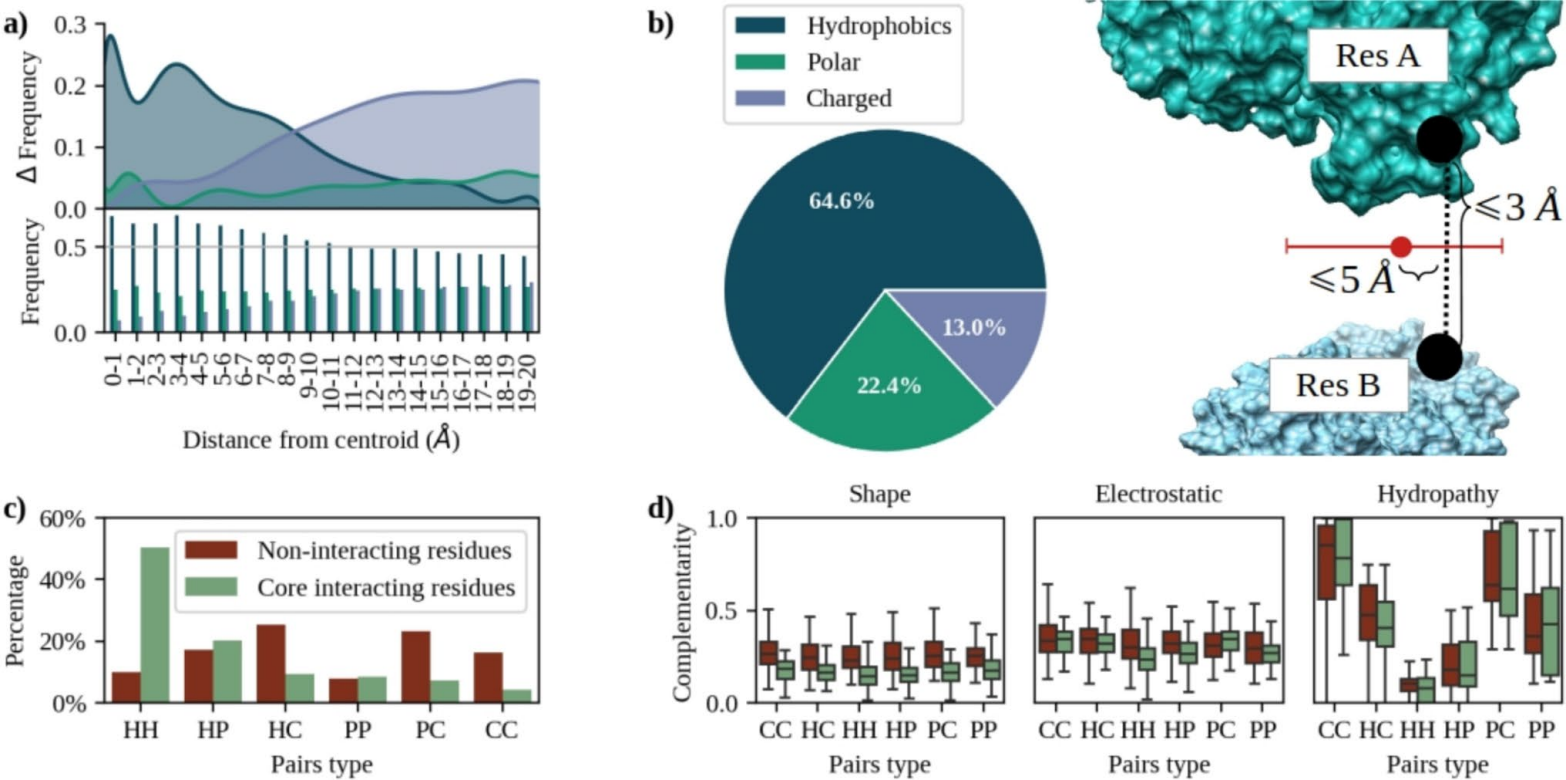
Characterization of core interacting residues pairs. **a** At the bottom, the frequency of each of the three amino acid classes -hydrophobic, polar, and charged (represented in dark blue, green, and light blue, respectively)- in 1 Å-wide annular regions at increasing distances from the center of the binding sites. At the top, the difference between the frequency in each annular region and the minimum frequency for that class. **b** On the left, the percentage of hydrophobic, polar, and charged residues found in core interacting pairs within the ‘Test dataset’. Core interacting pairs are those closer than 3 Å and within 5 Å from the interface center. **c** Frequency of each possible pairing between charged (C), polar (P), or hydrophobic (H) residues. Green bars indicate the frequency in core interacting residues, while red bars refer to the non-interacting residues found in the ‘Test dataset’. **d)** From left to right, shape, electrostatic, and hydropathy complementarity between core interacting residues (green) and non-interacting residues (red) in the ‘Test dataset’. Data are stratified based on the chemical nature of the residues, classified as charged, polar, or hydrophobic. All six possible pairings (CC, HP, PC, PP, HH, and HC) are considered

To evaluate how the three main features considered by CIRNet (shape, electrostatic, and hydropathy complementarity) behave at the interfaces, we selected from the complexes proposed by Gainza et al. (2020) the same balanced dataset we used to test CIRNet (Grassmann et al. 2024), counting 905 protein dimers with available structural data, that we here call ‘Test dataset’. To ensure a balanced set, we selected an equal number of patches centered on points sampled from interacting sites and randomly selected outside the interfaces (see Methods for more details). Interfaces are defined as the protein surface points within 6 Å of the partner surface. Since in the ‘Test dataset’ these regions have an average maximum radius of approximately 17 Å, we analyzed the contributions of amino acids within a 20 Å radius from the interfaces’ cores. To evaluate the chemical features of the regions surrounding the interface core, we divided them into annular regions with a 1 Å radius. Figure 1a confirms the well-known fact that amino acids in the interacting regions are mostly hydrophobic (H), especially near the center of the interfaces, but still contain a large fraction of polar (P) and charged (C) amino acids (Rego et al. 2021; Jones and Thornton 1996; Yan et al. 2008). The classification of the 20 natural amino acids in these three classes is reported in Table 1.

**Table 1:** Amino acids are classified as hydrophobics (H), polar (P), and charged (C)

H - Hydrophobics	
GLY, ALA, VAL, LEU, ILE, MET, PHE, TYR, TRP	

P - Polar	
SER, PRO, THR, CYS, ASN, GLN	

C - Charged	
HIS, LYS, ARG, ASP, GLU	

Up to a distance of approximately 10 Å from the interface centroid, hydrophobic residues oscillate between 50% and 70% of the total. At the rim of the binding sites, they become less abundant but remain the predominant class with a frequency of approximately 45%. Charged residues follow the opposite trend, increasing their frequency nearly six times at the rim of the binding site compared to its core. On the other hand, polar residues are uniformly distributed, with a mean frequency of approximately 25%.

These behaviors are reflected in the composition of the core interacting residue pairs in the ‘Test dataset’. Core pairs are those closer than 3 Å and within 5 Å from the interface center. These distances are computed taking the centroid of the surface points generated by the considered residues, and do not take into account water mediated contacts. As shown in Fig. 1b, most of these residues ($\sim$64%) are hydrophobic, while charged and polar amino acids are rare ($\sim$13% and $\sim$22% respectively). As shown in Fig. 1c, approximately 80% of core interacting residue pairs involve at least one H amino acid: in 51% of the cases both residues are hydrophobic (HH), 20% of the times the second residue is polar (HP), and in 9% of the cases charged (HC). Polar-polar pairings (PP) are $\sim$9% of the total, polar-charged (PC) $\sim$7%. Charged residues interacting with other charged residues are the most rare to observe at the core of interfaces, being only 4% of the total.

This suggests that CC pairs typically do not play a major role in establishing electrostatic complementarity at short distances within the regions analyzed in this study, where van der Waals interactions are maximized (Milanetti et al. 2021). Instead, it can be hypothesized that they are more involved in determining the well-known long-range effect of electrostatic interactions, guiding the initial recognition and approach of binding partners. This hypothesis is supported by Figs. 1a,b, showing that charged residues are rarely found at the core of the interface.

As expected, the solvent-exposed surface not involved in interaction does not present such a high concentration of hydrophobic amino acids. The non-interacting residues taken as decoys in the ‘Test dataset’ (red bars) have a more uniform distribution of the six possible pairings between amino acids. The residues in non-interacting pairs were randomly selected from those that did not generate any surface points within interacting regions and were then randomly paired.

The chemical composition of the interfaces is -by definition- reflected in the hydropathy complementarity values between core interacting residue pairs. We defined the hydropathy complementarity between two residues *A* and *B* as:

1 $$\begin{aligned} H_r = -a(H_A H_B)^2+b(H_AH_B), \end{aligned}$$

where $H_A$ and $H_B$ are the hydrophobicity indices of the two amino acids. Hydrophobicity indices close to zero are assigned to hydrophobic residues, while higher values indicate more hydrophilic amino acids. The parameters *a* and *b* are set to 0.033 and 0.363 respectively, so that $H_r$ is defined as a parabolic function with roots corresponding to the minimum and maximum products of the hydrophobicity indices, and vertex at one. Thus, $H_r$ values close to zero correspond to higher hydropathy complementarity, as residues with similar *H* values have stronger interactions than residues with opposing hydropathy characteristics.

This is shown in Fig. 1d, where the best hydropathy complementarity (low $H_r$) values are obtained for HH pairs. These bonds are energetically favorable since they reduce the exposure of hydrophobic surfaces to the aqueous environment. The same panel also shows that hydropathy complementarity can distinguish to a certain level between core interacting and non-interacting residues: the distributions of the hydropathy complementarity values computed between core interacting and non interacting residues pairs are distinguished with a mean ROC AUC of $\sim$0.7 across the six pairs types.

To study the behavior of shape and electrostatic complementarity, for each complex we computed the solvent-accessible surface and the associated electrostatic potential surface (as described in the Methods Section), and then evaluated the shape and electrostatic complementarity between regions with the Zernike formalism (see Methods for more details). Figure 1d shows that stratifying these two features according to the C, P, or H nature of the involved residues does not result in appreciable variation. The ROC AUC values between the shape complementarity distribution of core interacting residues from different chemical classes oscillate between 0.51 and 0.61. Even electrostatic complementarity is mostly independent of the residue pairs, with the most noticeable exception being the distribution of HH core interacting pairs when compared to those of HC and PC residues (ROC AUC of $\sim$0.75 and $\sim$0.79 respectively).

Electrostatic complementarity has a similar performance compared to hydropathy complementarity in distinguishing core interacting and non-interacting residues, as testified by a mean ROC AUC of 0.65. On the other hand, the distribution of shape complementarity between core interacting residues is shifted on a lower range compared to that of non-interacting residues, resulting in a mean ROC AUC of 0.8.

These observations confirm the well-known fact that binding regions tend to have a hydrophobic core and a charged rim, and are characterized by a higher level of shape complementarity compared to random decoys (Grassmann et al. 2024). Electrostatic and hydropathy complementarity also show a promising variation among core interacting and non-interacting residues, and a variation in distribution for different chemical classes of residue pairs.

### Shape and hydropathy complementarity bring the most predictive information

Next, we studied the role of shape, electrostatic, and hydropathy complementarity and their interplay. In particular, we analyzed the input space investigated by CIRNet and how our NN reconstructs it.

**Fig. 2:**
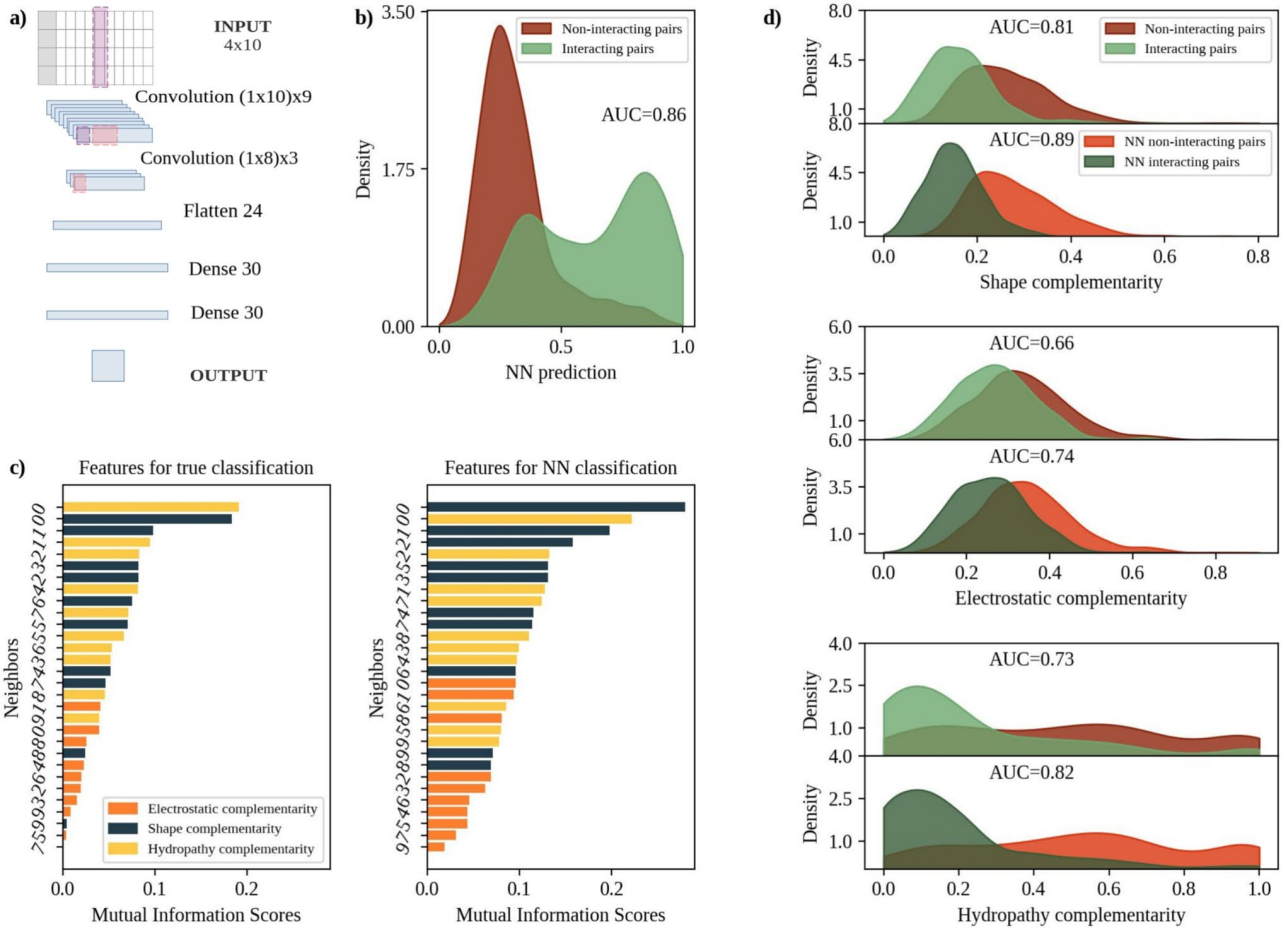
Most relevant chemico-physical features for CIRNet training. **a** Each residue pair is associated with a 4$\times$10 matrix that summarizes the shape, electrostatic, and hydropathy complementarity levels between the first analyzed residue and the neighborhood of the second one. This matrix is fed into CIRNet, a NN with two convolutional and two dense layers. CIRNet then classifies each residue pair based on its probability of being at the core of an interface. **b** At the top, NN classifications from the ‘Test dataset’ are divided into true core interacting residues (green) and decoys (red). The bottom panel shows the ROC curve for these classifications, with the AUC value displayed. **c** Features characterizing residue pairs are ranked according to their Mutual Information Score contribution to true classification (left) and NN classification (right). Each feature represents complementarity of shape, electrostatics, or hydropathy (in blue, orange, and yellow, respectively) between a residue and its putative partner (neighbor 0) or one of its partner’s neighbors, as indicated on the y-axis. **d** From top to bottom, the distributions of shape, electrostatic, and hydropathy complementarity between residue pairs in the ‘Test dataset’. Complementarity values are divided into true (top) and NN-predicted (bottom) non-interacting and interacting pairs, shown in red and green hues, respectively. For each case, the ROC AUC between interacting and non-interacting distributions is reported

In addition to the shape, electrostatic, and hydropathy complementarity between the two putative core interacting residues, CIRNet receives the complementarities between the first residue and the first nine neighbors of the second amino acid (the architecture of CIRNet is shown in Fig. 2a).

Relying on these features, CIRNet achieves a ROC AUC of 0.86 in distinguishing between core interacting residues and random decoys from the ‘Test dataset’, as shown in Fig. 2b. The prediction threshold for the NN is fixed at 0.38, which maximizes the True Positive Rate while minimizing the False Positive Rate.

To measure the amount of information of each feature we compute the mutual information (MI) score between each of the thirty features and the real classification of residue pairs as core interacting or not (see Methods for more details). The MI between two variables measures their mutual dependence by measuring the amount of information one variable provides about the other: values close to one indicate stronger connections between the feature and the target. The left panel of Fig. 2c shows that the highest MI is obtained for the hydropathy and shape complementarity between the putative core interacting residues (MI close to 0.2). The complementarities computed against the neighbors of the considered residue still define core interacting residues, especially when compared to the electrostatic complementarity, for which the maximum MI has a value of 0.04. The right panel of Fig. 2c reports the MI scores computed against the NN prediction. It can be seen that CIRNet mostly reconstructs the information rank of the different features. Electrostatic complementarity is the least informative feature, while the hydropathy and shape complementarity between the putative core interacting residues still show the highest MI (0.22 and 0.28 respectively). This panel suggests that CIRNet relies strongly on shape and electrostatic complementarity, as shown by MI values that are higher compared to the top panel: the average MI of the first three features has a value of 0.16 and 0.23 for the true and NN classification, respectively.

Figure 2d confirms that the representation chosen by CIRNet relies heavily on the level of complementarity among residues. Stratifying the values of shape, electrostatic, and hydropathy complementarity of the considered residue pairs according to the true classification or the NN prediction shows that in the second case, the two distributions are more separated. For shape, electrostatic, and hydropathy complementarity the ROC AUC is increased from 0.8 to 0.89, from 0.66 to 0.73, and from 0.73 to 0.82, respectively.

CIRNet expects interacting residues to be associated with high complementarities (corresponding to low numerical values of the features that it receives as input). In particular, it focuses on shape and hydropathy complementarity. This results in a wrong classification of residue pairs that are characterized by high level of complementarity despite being non-interacting.

We next checked if there is a clear interplay between these features and if CIRNet can pick it up.

### Electrostatic complementarity plays an important role in the data representation

**Fig. 3:**
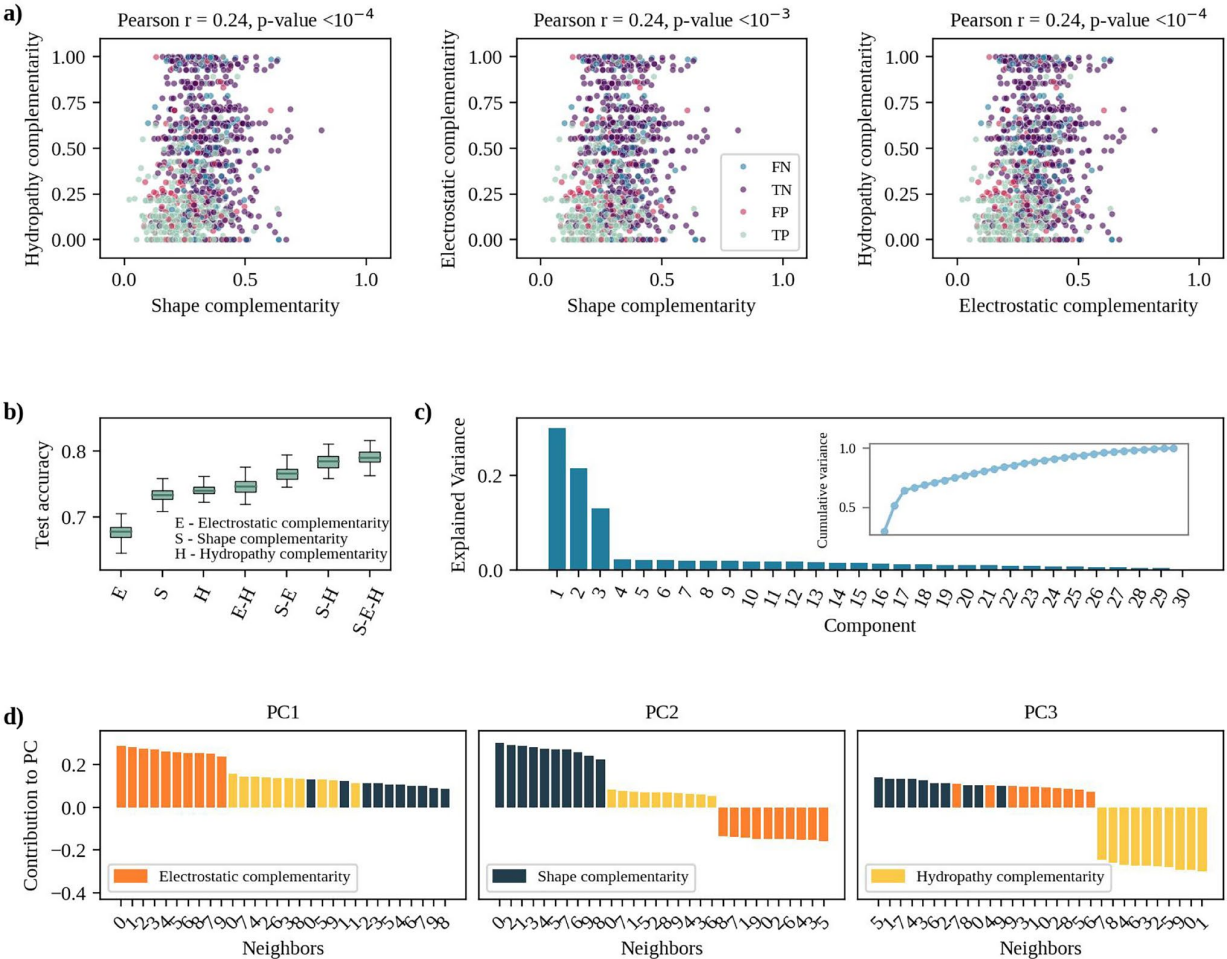
Principal component analysis of the CIRNet input observables. **a** Hydropathy complementarity (left plot) and electrostatic complementarity (center plot) as functions of shape complementarity. The right plot shows electrostatic complementarity as a function of hydropathy complementarity. Each point corresponds to one of the residue pairs in the ‘Test dataset’ and is colored according to NN-predicted interaction. Blue (pink) dots indicate false negatives (positives), while green (violet) represent true negatives (positives). **b** For each features subset, CIRNet is trained and tested on the ’CIRNet dataset’ for 100 repetitions. The accuracies resulting from each testing are reported in the box-plot. **c** Explained variance of each of the principal components describing the ‘Test dataset’. In the insert, the cumulative variance obtained for each added component. **d** Each bar represents the contribution of one of the 30 original features to PC1, PC2, and PC3 (from left to right). Each feature measures complementarity in terms of shape, electrostatics, or hydropathy (shown in yellow, red, and orange, respectively) between a residue and its putative binding partner or one of its neighbors

We then investigated the interplay between shape, electrostatic, and hydropathy complementarity. Figure 3a shows that their relations are not described by simple linear correlations. Hydropathy complementarity has a Pearson correlation with shape complementarity and electrostatic complementarity of 0.25 (p-value$=2.66\times 10^{-19}$) and 0.24 (p-value$=1.66\times 10^{-18}$), respectively. Shape and electrostatic complementarity have a weaker correlation of 0.10 (p-value$=1.69\times 10^{-4}$). To clarify the dependency between the features, as a first step we trained and tested CIRNet on the ‘CIRNet dataset’ (see Methods for more details) using 100 repetitions of all the possible subsets of features. Figure 3b shows that using electrostatic, shape, or hydropathy complementarity alone results in lower testing accuracy. This is due to a degree of degeneracy in the characterization of surface patches when each feature is considered in isolation. For example, small flat regions, commonly found across protein surfaces, exhibit high shape complementarity despite being non-interacting. Incorporating additional features, such as electrostatic and hydropathy complementarity, enables a more precise characterization, allowing for a more accurate selection of binding regions. From Fig. 3b it can be seen that electrostatic complementarity alone results in the worst performance, reaching a mean test accuracy of $\sim$0.68. Shape and hydropathy complementarity instead -even when not merged to other features- result in a mean accuracy of $\sim$0.73 and $\sim$0.74 respectively. Electrostatic merged to shape complementarity improves the performance to $\sim$0.76, while when added to hydropathy complementarity it does not improve significantly the accuracy. Even if the three features together results in the highest mean accuracy ($\sim$0.79), shape and hydropathy complementarity without electrostatic produce a mean value of $\sim$0.78. As already observed with the MI score, these are the features bringing the most predictive information for the NN prediction.

To test if electrostatic complementarity nevertheless has a role in the network learning, we studied which features dominate the whole dataset’s variance structure with a Principal Component Analysis (PCA), shown in Fig. 3d. By computing the Explained Variance Ratio (EVR) of each component (see Methods for more details), we observed that the first three components (ordered according to their eigenvalues) can be identified as the Principal Components (PCs) according to the Scree test (Jolliffe 2002), reaching a total cumulative variance of $\sim$0.64. We then analyzed how each of the thirty features contributes to the selected PCs (PC1, PC2, and PC3) by looking at their loadings (i.e., the coefficients of the linear combination of the original variables constructing the PCs). Figure 3d shows that the strongest contributions to PC1 come from electrostatic complementarity, but shape and hydropathy complementarity appear in a similar proportion. Shape complementarity also determines PC2 and PC3. The absence of low-variance or correlated features, suggest that the dataset can not be further simplified and that all the implemented input data are used by the network.

### CIRNet prediction reflects the different chemical classes of residue pairs

We verified that CIRNet extracts as much information as possible when taking the input data and relies not only on shape and hydropathy complementarity (having the most predictive information) but also on electrostatic complementarity. Having observed that electrostatic complementarity is differently distributed for the six chemical classes of residue pairs (see Fig. 1), we investigated the chemical significance of the network’s learning. Specifically, we stratified the classification of pairs from the ‘Test dataset’ based on residue types (hydrophobic, charged, and polar).

**Fig. 4:**
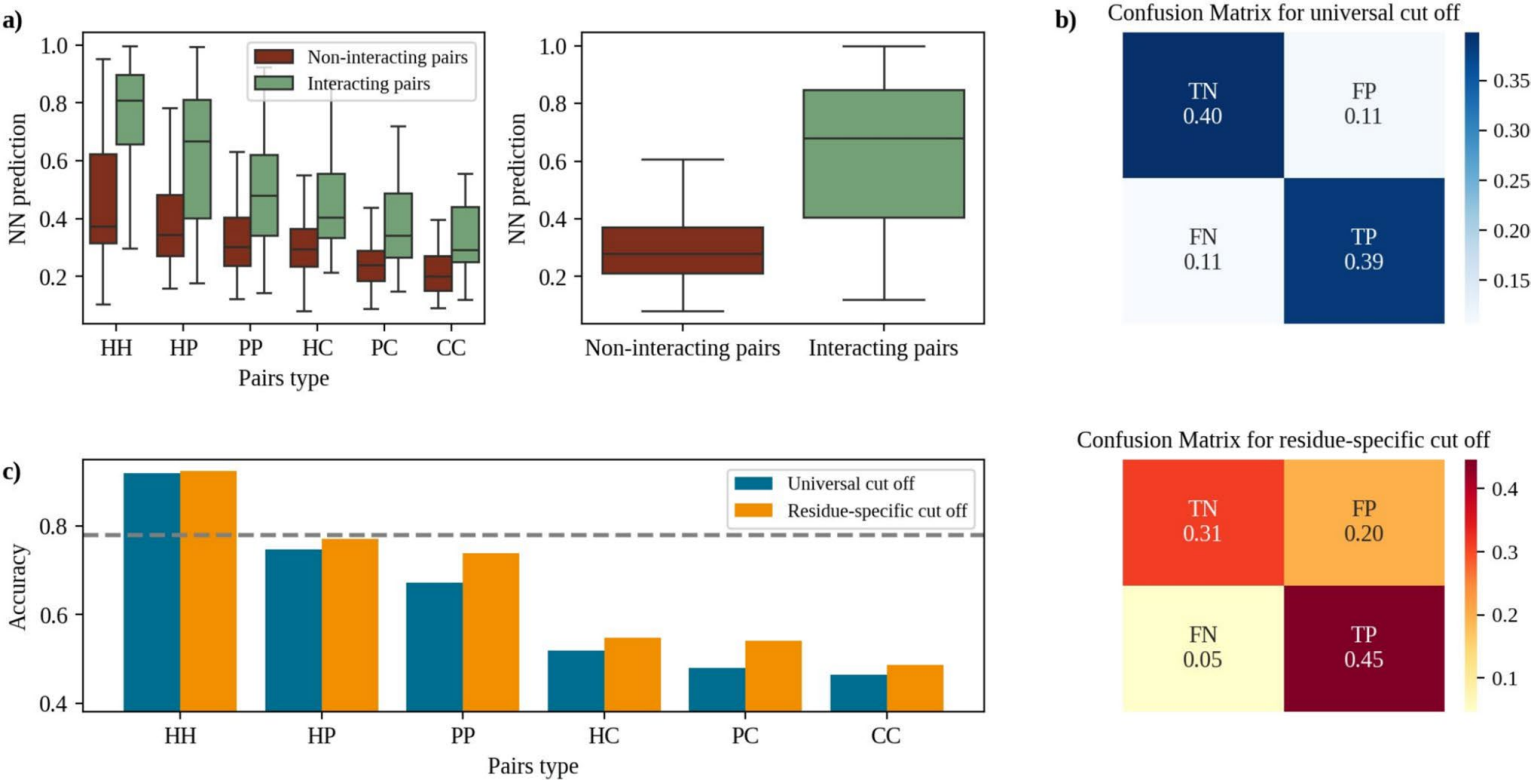
Residue-specific cut-offs impact on interaction prediction **a** On the left, the distributions from Fig. 2b are further stratified by the chemical nature of the residues: charged (C), polar (P), or hydrophobic (H). The six pairings are organized based on their mean NN prediction values. On the right, the distributions from Fig. 2b are reported in a box plot to facilitate the comparison. **b** On top, the confusion matrix was obtained using the universal cut-off of 0.38 to define residues as core interacting pairs based on the NN prediction value. On the bottom, the confusion matrix was obtained when using the residue-specific cut-off reported in Table 2. **c** The blue columns show the F1-score obtained for each residue pair class using the universal threshold. The orange columns show the F1-score obtained with the threshold computed specifically for that class. The gray line indicates the F1-score computed over the whole dataset

Figure 4a shows that the NN-predicted distinction between interacting and non-interacting pairs is clearest for HH and HP, where there is minimal overlap in distributions. For other types, particularly PP and CC, there is more overlap, suggesting the model might struggle to differentiate interactions here. This is confirmed by the ROC AUC reported in Table 2. The comparison between the left and right panels of Fig. 4a suggests the introduction of residue-specific thresholds since each class is shifted to a different range of values. Figure 4b shows that for the already discussed universal cut-off of 0.38, the true positive rate and true negative rate are balanced (0.39 and 0.4 respectively). On the other hand, the false positive rate (0.11) and false negative rate (0.11) indicate some misclassifications on both sides. When adopting the residue-specific thresholds reported in Table 2, the true positive rate improves significantly (0.45), suggesting better detection of positives with residue-specific adjustments. The false negative rate drops (0.05), indicating fewer missed positive predictions. On the other hand, the true negative rate decreases (0.31), with an increase in false positives (0.20), showing a slight trade-off in specificity for improved sensitivity.

Figure 4c shows that CIRNet’s performance declines when at least one of the residues is charged. We hypothesize that this is because charged residues are less frequently found in core interacting pairs (see Fig. 1b) than in random decoys, as they are more often located on the solvent-exposed surface rather than in the core of the interfaces (see Fig. 1a). Expanding the dataset could improve CIRNet’s accuracy by providing more examples of charged residues during training. However, we selected the largest available dataset-comprising protein complexes with structural data obtained via X-ray crystallography and known experimental pH values (see Methods for more details) (Grassmann et al. 2024).

When looking at the accuracy for each class, Fig. 4c shows that using specific thresholds brings improvements, especially for PC and PP pairs, whose F1-scores increase by 12% and 10% respectively.

These observations suggest the possibility of a future version of CIRNet with enhanced accuracy achieved by incorporating a label in the input data that reflects the chemical nature of the residues.

**Table 2:** Residue-specific threshold. Thresholds maximizing the separation between the distributions of interacting and non-interacting residues, in terms of F1-score, stratified by residue type

Pairs type	ROC AUC	Threshold
HH	0.83	0.26
HP	0.79	0.32
PP	0.73	0.31
HC	0.75	0.36
PC	0.77	0.28
CC	0.76	0.27

## Conclusions

Since over 80% of proteins operate in molecular complexes (Srinivasa Rao et al. 2014), understanding the molecular mechanisms regulating protein-protein interactions and predicting protein complexes is fundamental, with important implications in many applied fields such as protein design and the mapping of the human interactome (Zhang et al. 2012; Hermann et al. 2007; Kortemme et al. 2004; Miotto et al. 2024).

Machine learning-based approaches have brought big advancements in this field, as tested in the latest editions of the CAPRI competition (Joël et al. 2003). In 2022, during CAPRI Round 54, high-quality models were produced for about 40% of the targets compared to 8% two years earlier (Lensink et al. 2023). Nevertheless, the identification and characterization of interfaces remain a challenge: even a successful tool such as AlphaFold 3 (Abramson et al. 2024) still has some limitations, such as producing clashing atoms and hallucinations in disordered regions. Moreover, it relies on multiple sequence alignments and its choices lack a clear physical-chemical interpretation: it does not give insight into the real mechanisms at the core of protein interactions.

The first step to address this problem, shared by many machine learning approaches, is identifying the features at the core of protein binding and characterize their interplay. Thus, deciphering the internals of machine learning tools could bring a new light on the prediction of protein interactions.

With this aim, we here analyzed the information space of CIRNet, a neural network we recently developed to identify pairs of interacting residues at the core of protein binding regions (Grassmann et al. 2024). We showed that CIRNet can identify these pairs mainly by searching for pairs with the highest level of shape, electrostatic, and hydropathy complementarity. Shape and hydropathy complementarity bring the most predictive information, but electrostatic complementarity still shows a significant contribution to the definition of the dataset. We propose two possible explanations: (i) shape and hydropathy complementarity play a more decisive role than electrostatic complementarity in all protein-protein interfaces, or (ii) while shape and hydropathy complementarity are predominant in most binding processes, electrostatic complementarity becomes more significant in specific cases. This insight paves the way for future investigations into the various mechanisms by which binding partners achieve binding specificity.

Merging all these features and capturing the interplay between them, CIRNet distinguishes between interacting and non-interacting pairs with a ROC AUC of 0.86. Furthermore, we observed that the NN prediction can be stratified according to the chemical class of the amino acids. Thus, we hypothesize that an advanced version of CIRNet could be obtained by explicitly taking into account the chemical class of the residues in the input data.

## Methods

### Dataset

To probe the information brought from the different physico-chemical features, we analyzed a dataset of 905 protein-protein dimers used to test CIRNet (Grassmann et al. 2024), which we refer to here as ‘Test dataset’.

In the part of the study focused on assessing CIRNet’s performance variations with different combinations of the original features, we used the complete dataset employed to train, validate, and test CIRNet (Grassmann et al. 2024). This dataset, which we call the ‘CIRNet dataset’, contains 3,621 complexes, including those from the ‘Test dataset’.

This dataset, extracted from the one used by Milanetti et al. (2021), includes complexes with structural data obtained via X-ray crystallography, sourced from the 3D Complex Database (Levy et al. 2006), and features known experimental pH values.

It is important to note that approximately 60% of the complexes in this dataset display core interacting residues at their interfaces. We included complexes lacking this feature in the dataset to evaluate CIRNet performance across different types of complexes (Grassmann et al. 2024).

### Computation of the surfaces and binding sites definition

For each protein in the dataset, we computed the solvent-accessible surface with DMS (Richards 1977), using a resolution of 5 points per Å$^2$ and a water probe radius of 1.4 Å.

To define the electrostatic potential surface, we started by calculating the electrostatic potential with the APBS code (Jurrus et al. 2017), considering the experimental pH and treating each protein independently from its partner. Next, we created a grid and assigned to each cell the electrostatic potential values corresponding to the surface points included in that cell.

Finally, we defined binding sites as the regions on a protein surface within 6 Å of the partner surface. To determine the center of an interface, we calculated the centroid of the surface points within that binding region.

### Patch definition and projection

To define a surface patch, we center a sphere with a radius of $R=$9Å on a surface point and select the surface points within this sphere. Previous studies (Milanetti et al. 2021; Grassmann et al. 2023) have shown that this *R* value results in an optimal identification of binding regions based on shape and electrostatic complementarity.

As a next step, we fit a plane to the selected patch points and re-orient the patch so that its normal vector is perpendicular to the x-y plane. To evaluate the shape complementarity between two patches, they must be re-oriented so that their solvent-exposed sides face opposite verses on the z-axis.

Each patch point is associated with its three spatial coordinates and the electrostatic potential in that region. After the re-orientation, these descriptions are projected onto the x-y plane.

With this aim, we define a point *C* on the z-axis such that the angle $\theta$ between the z-axis and the secants connecting *C* to any point on the patch are less than $\theta =45^\circ$. Each patch point is labeled with its distance *r* from *C*. A 25$\times$25 pixels grid is constructed, and each pixel is associated with the mean *r* value calculated on the points projected inside it. This results in the so-called shape projection.

For the electrostatic projection, we create a separate 25$\times$25 grid and assign to each pixel the mean electrostatic potential of the points projected in it.

The parameters used for these projections were identified as the most effective by Milanetti et al. (2021).

### Zernike 2D protocol

The shape and electrostatic projections of a surface patch are functions of two variables $f(r,\psi )$ defined in polar coordinates inside the region of the unitary circle. These functions can be decomposed in the Zernike basis:

2 $$\begin{aligned} f(r,\psi )=\sum _{n'=0}^\infty \sum _{m=0}^{n'}c_{n'm}Z_{n'm}(r,\psi ), \end{aligned}$$

where

3 $$\begin{aligned} c_{n'm}=\frac{n'+1}{\pi }\int _0^1dr~r\int _0^{2\pi }d\psi Z_{n'm}^*(r,\psi )f(r,\psi ) \end{aligned}$$

and

4 $$\begin{aligned} Z_{n'm}=R_{n'm}(r)e^{im\psi }. \end{aligned}$$

$c_{n'm}$ are the expansion coefficients, while the complex functions $Z_{n'm}(r,\psi )$ are the Zernike polynomials. The radial part $R_{n'm}$ is given by

5 $$\begin{aligned} R_{n'm}(r)=\sum _{k=0}^{\frac{n'-m}{2}}\frac{(-1)^k(n'-k)!}{k!\big (\frac{n'+m}{2}-k\big )!\big (\frac{n'-m}{2}-k\big )!}. \end{aligned}$$

The set of complex expansion coefficients ${c_{n'm}}$ uniquely reconstructs the original function, with a resolution given by the order of expansion $N=max(n^{'})$. The norms of these expansion coefficients are known as Zernike descriptors and are invariant to rotations around the origin of the unit circle.

By describing two functions with their Zernike invariant vectors, we can quantify their similarity by computing the Euclidean distance between the Zernike vectors. For our shape and electrostatic projections, a smaller distance between the Zernike vectors of two patches indicates higher complementarity (since one of the patches is inverted in this comparison) (Milanetti et al. 2021; Grassmann et al. 2023).

We used $R=9$Å and $N=20$, identified as the most effective parameters in a previous work (Milanetti et al. 2021). Smaller values of *R* result in patches that are too small to adequately distinguish interactions. A larger radius would include non-interacting regions that inherently have low complementarity. Lower values of *N* lead to excessively "smooth" reconstructions, while excessively high values capture unnecessary and time-consuming details.

### Hydrophobicity scale

Di Rienzo et al. (2021) proposed a hydrophobicity scale based on MD simulations for each of the 20 natural amino acids. They analyzed the variations in the hydrogen bond network of the surrounding water molecules and observed the spatial reorganization in the local structure. This method allowed them to evaluate the hydrophilicity and hydrophobicity features of each amino acid and associate it with a hydrophobicity index *H*. The resulting hydrophobicity scale, which considers the collective response of protein hydration waters to the local nanoscale chemical and topographical patterns, allows a compact representation and comparison of the hydrophobicity of molecular surface patches.

### Neural network architecture

The neural network analyzed in this study, CIRNet, was introduced in a previous work (Grassmann et al. 2024). It consists of two convolutional layers (Krizhevsky et al. 2017) followed by two dense layers. The convolutional layers are followed by a dropout layer, to prevent overfitting. Next, the learned features are flattened into a 1D vector and passed through two fully connected layers. The final output layer predicts the probability that the input residue pair is a core interacting pair. Core interacting pairs are those whose residues are within 3 Å from each other and less than 5 Å from the interface center.

CIRNet receives as input, for each residue pair, a $4\times 10$ matrix that represents the normalized complementarity of the shape, electrostatic, and hydropathy complementarity between the first residue and a 10 Å radius region centered on the second amino acid. To associate to each residue pair a single value for each kind of complementarity, we computed the mean values of the $Z_s$ (shape complementarity) and $Z_{el}$ (electrostatic complementarity) between the patches centered on the surface points generated by that amino acids. The $H_r$ (hydropathy complementarity) value was calculated starting from the *H* value of each residue in the pair.

### Mutual information score

The MI between two variables *X*, *Y* (in our case input feature and output or classification data) is the relative entropy between their joint distribution and the product of their marginal distributions:

6 $$\begin{aligned} MI(X,Y) = \sum _X \sum _Y p(x,y) log\Big (\frac{p(x,y)}{p(x)p(y)}\Big ), \end{aligned}$$

where *p*(*x*, *y*) is the joint probability of the two variables, and *p*(*x*), *p*(*y*) are their marginal probabilities. Higher values (close to one) indicate a closer connection between the variables.

### Principal Component Analysis and Explained Variance

PCA is a multivariate statistics technique that reduces the degrees of freedom in a dataset. With this aim, the basis vector describing the data is transformed into an orthogonal basis composed of the eigenvectors of the covariance matrix $\hat{C}$ for the set of observables. These are the so-called Principal Components (PCs). The PCs are then sorted in order of decreasing values of the corresponding eigenvalues, reflecting the information they bring about the variability of the data. The reduction of degrees of freedom is obtained by projecting the dataset features into a subset of this basis defined by the first *d* PCs.

A quantitative estimate of the information collected by each PC corresponding to an eigenvalue $\lambda _i$, is given by the EVR:

7 $$\begin{aligned} EVR(\lambda _i)=\frac{\lambda _i}{\sum _j^{N} \lambda _j}, \end{aligned}$$

where *N* is the number of original features.

## Data Availability

All relevant data are displayed within the manuscript. Raw data can be requested to the corresponding authors.
